# Abdominal Obesity: An Independent Influencing Factor of Visuospatial and Executive/Language Ability and the Serum Levels of A*β*40/A*β*42/Tau Protein

**DOI:** 10.1155/2022/3622149

**Published:** 2022-04-01

**Authors:** Xin Fan, Yun Zhong, Lingling Zhang, Jiaqi Li, Fei Xie, Zhiyuan Zhang

**Affiliations:** ^1^Department of Otolaryngology Head and Neck Surgery, The First Affiliated Hospital of Nanchang University, Nanchang 330000, China; ^2^The First Clinical Medical College of Nanchang University, Nanchang 330000, China; ^3^School of Stomatology, Nanchang University, Nanchang 330000, China

## Abstract

**Background:**

Although obesity affects human health and cognitive function, the influence of abdominal obesity on cognitive function is still unclear.

**Methods:**

The MoCA scale was used to evaluate the overall cognitive function and the function of each subitem of 196 subjects, as well as the SDMT and TMT-A scales for evaluating the attention and information processing speed. In addition, radioimmunoassay was used to detect the serum levels of A*β*40, A*β*42, and tau protein in 45 subjects. Subjects were divided into abdominal and nonabdominal obesity groups. Before and after correcting confounding factors, the differences in cognitive scale evaluation indexes and three protein levels between the two groups were compared. We also explore further the correlation between various cognitive abilities and the waist circumference/levels of the three proteins. Linear regression was used to identify the independent influencing factors of various cognitive functions and three protein levels.

**Results:**

After correcting for multiple factors, we observed the lower scores of visuospatial function, execution, and language in the MoCA scale, as well as higher levels of A*β*40 and tau protein in the abdominal obesity group, supported by the results of correlation analysis. Abdominal obesity was identified as an independent negative influencing factor of MoCA visual space, executive power, and language scores and an independent positive influencing factor of A*β*40, A*β*42, and tau protein levels.

**Conclusion:**

Abdominal obesity may play a negative role in visuospatial, executive ability, and language function and a positive role in the A*β*40, A*β*42, and tau protein serum levels.

## 1. Introduction

The pathological manifestations caused by excessive fat content or abnormal fat distribution in the human body are called obesity. Obesity is becoming more and more common worldwide. Between 1980 and 2015, the number of obese children and adults in 73 countries doubled [1]. In 2016, more than 1.9 billion adults, 18 years and older, were overweight. Of these, over 650 million were obese [2]. Projections for 2022 are that the prevalence of obesity may reach 24.8% [3]. The China Health and Nutrition Survey (CHNS) showed that between 1993 and 2009, the prevalence of adult overweight/obesity increased from 13.4% to 26.4%, and the prevalence of adult abdominal obesity increased from 18.6% to 37.4%, which reveals that the prevalence of abdominal obesity is increasing faster than overweight/obesity [4]. Related studies have also found that the body fat distribution of Asian populations is more inclined to abdominal obesity [5].

Obesity is a recognized risk factor for various chronic physical health diseases, including metabolic syndrome, hypertension, cardiovascular disease, diabetes, stroke, and cancer [6–9]. Compared with general obesity, abdominal obesity has a stronger connection with these chronic diseases [10, 11]. These chronic diseases caused by abdominal obesity have complex and diverse causes, high prevalence, and a long course of the disease, which have also produced a huge burden of disease. Abdominal obesity has become one of the public problems that Chinese adults urgently need to solve.

In addition, obesity is also inextricably linked with neurodegenerative diseases. But the results of research on the effects of obesity on these diseases appear to be ambiguous. For example, some studies have found that obesity can lead to cognitive dysfunction [12, 13], and have identified obesity as an independent influence factor of cognitive function [14]. Studies have also found that obesity is associated with an increased risk of dementia [15]. However, some recent studies failed to find evidence that obesity increases the risk of dementia [16, 17]. Other studies have even found that underweight people are at greater risk, thus intuitively regard obesity as a protective factor for dementia [18, 19]. At present, the measurement effect of body mass index (BMI) on obesity has been proven to be inferior to waist circumference (WC) and waist-to-hip ratio (WHR) by more and more studies [20]. Chelsea et al. also believe that BMI is not a highly sensitive measurement method because it cannot distinguish between fat mass and lean body mass nor does fat distribution. As an alternative measure of obesity, WC can be used to define abdominal obesity and be used in future research related to cognitive function [21]. Therefore, the divergent conclusions about the influence of obesity on cognitive function may be caused by the widespread use of BMI to define obesity. At the same time, the excessive dependence on cross-sectional design and the lack of specificity in assessing the basic areas also contributed to the mixed findings of the impact of obesity on cognition [22]. A few studies have found that abdominal obesity defined by the WHR cut-off value is significantly associated with the risk of cognitive impairment [23, 24]. However, related research on abdominal obesity defined by WC is still lacking.

Alzheimer disease (AD) is a chronic neurodegenerative disease. Its main symptoms include gradual memory decline, cognitive function, and behavioral disorders such as learning, language, and spatial orientation [25]. The typical pathological features of AD are the accumulation of amyloid *β*-protein (A*β*) outside the cell and excessive tau protein, which leads to amyloid plaques, neurofibrillary tangles, and neuronal apoptosis [26]. Eventually, these lead to cognitive dysfunction. At present, various literature reports have confirmed that A*β*42 in the cerebrospinal fluid of AD patients is increased, and the phosphorylated tau protein is increased. Similarly, Mild Cognitive Impairment (MCI), as a transitional precursor stage from normal aging to dementia [27], has also been linked to the pathological damage mechanism of A*β* and tau protein in many studies. Although obesity is considered an influencing factor of AD [28], there are currently few human studies on the relationship between abdominal obesity/cognitive function of patients with abdominal obesity and A*β* and tau protein [29–31].

Chinese-Beijing Version of Montreal Cognitive Assessment (MoCA-C) is a widely used method to measure cognitive function in Chinese [32]. The Symbol Digit Modalities Test (SDMT) [33, 34] and Trail Making Test-Part A (TMT-A) [35, 36] are two representative tools for assessing general cognitive function. This study is aimed at exploring the effects of abdominal obesity on cognitive function indexes assessed by the MoCA-C, SDMT, and TMT-A scale and the serum levels of A*β*40, A*β*42, and tau protein and the correlation among the three and finally exploring the potential mechanism of action between them.

## 2. Methods

### 2.1. Ethical Approval and Participants

This study was approved by the ethics committee of the First Affiliated Hospital of Nanchang University (approval number: 2019-05-051) and was carried out following the Declaration of Helsinki. Subjects were informed of the general content of the study before taking the test, followed the principle of voluntariness, and signed the informed consent form. The inclusion and exclusion criteria of participants are as follows:

Inclusion criteria: (1) the age group being 18-72 years old, (2) the number of years of education ≥ 5 years, (3) being able to fully understand and sign the informed consent form voluntarily, and (4) being able to independently complete tests of various cognitive scales.

Exclusion criteria: (1) having a history of cardiovascular complication severely affecting the body (such as heart failure, severe cerebral infarction, and myocardial infarction), (2) having a history of mental or neurological diseases or a history of psychotropic drug dependence, (3) recently affected the history of neurological brain drugs, (4) drinking alcoholic beverages within 24 hours before receiving relevant tests, (5) being unable to cooperate with the research due to various reasons, (6) missing or incomplete data, (7) pregnant or breastfeeding women, (8) recent major surgery, (9) having the history of participating in clinical research on weight loss or any other weight loss therapy in the past three months, (10) other diseases or reasons unable to cooperate with the research.

### 2.2. Basic and Human Data Collection

Subject's basic information (gender and age), disease history (diabetes and hypertension), smoking history, and alcohol intake history were collected.

Height, weight, neck circumference (NC), waist circumference (WC), hip circumference (HC), and BMI are measured and assigned readings by the same professional researchers using the same measuring equipment (tape measure, automatic height and weight instrument), based on the WHO standard method. According to the criteria for determining abdominal obesity of Chinese adults: WC ≥ 90 cm for men and WC ≥ 85 cm for women [37].

To reduce the influence of obstructive sleep apnea-hypopnea syndrome (OSAHS) on the final result, Polysomnography (PSG) from professional measurement was always performed at 10 PM and concluded at 6 AM the following day. apnea-hypopnea index (AHI), oxygen-desaturation index (ODI), lowest oxygen saturation (LSpO2) obtained by PSG were used in this study. According to the standards set by the American Sleep Medicine Association, AHI ≥30 is defined as severe OSAHS [38, 39].

### 2.3. Cognitive Data Collection

Participants in the study are not allowed to drink strong tea, wine, and coffee within 24 hours and must not have a history of taking sedatives and hypnotic drugs soon. All research results are conducted under the same standard guidance by the same professionally trained personnel. After completing the assessment, the same person analyzes each field's scores and total scores according to the same standards.

Cognitive data can be used for the following neuropsychological tests:

1. Epworth Sleepiness Scale (ESS): it was used to assess subjective sleepiness [40]
2. MoCA-C: it includes seven areas of visuospatial and executive, naming, attention, language, abstraction, delayed recall, and orientation [32]
3. SDMT: referring to a key at the top of the page to translate nonverbal symbols to an alpha-numeric digit, participant filled in boxes (written version) or verbalized the correct digit for each symbol on this timed test. Total correct responses within 90 seconds were measured [33, 34]
4. TMT-A: this requires an individual to sequence numbers within the format of a visual motor task. This measures processing speed [35, 36].

### 2.4. Blood Collection, Processing, and Protein Level Determination

After the PSG monitoring, 5 ml of peripheral fasting venous blood was drawn from the subject and centrifuged at 3000 r/min for 10 min within 60 min. The separated serum was stored in a cryotube and immediately frozen at -80°C until a batch determination was performed. Radioimmunoassay (RIA) was used to detect the levels of A*β*40, A*β*42, and tau protein. The operation was carried out in strict accordance with the instructions of the kit (Shanghai Haling Biological Technology Co., Ltd.).

### 2.5. Statistical Analysis

Use SPSS22.0 software to perform statistical analysis on the data. The measurement data is expressed in terms of *X* ± *S*, and the counting data is expressed in frequency and/or percentage. Continuous variables (baseline data such as gender, PSG indicators such as ODI between the two groups of abdominal obesity) used Mann-Whitney *U* test or *t*-test according to the distribution characteristics. When comparing the cognitive function assessment indicators and the three protein levels between the two groups, the Mann-Whitney *U* test or *t*-test was used when no factor was corrected. The covariance analysis was used when multiple factors were corrected. The corresponding mulberry diagram is drawn by GraphPad prism 9.0.0 (GraphPad Software, La Jolla, California, USA). Categorical variables (including gender, smoking, alcohol consumption history, etc.) use the Chi-square test/Fisher's exact test. Use multiple linear regression analysis to determine the factors affecting cognitive function and the level of each protein content. Spearman's rank correlation analysis between WC and various cognitive function scores, between WC and A*β*40, A*β*42, and tau protein levels, and between various cognitive functions and A*β*40, A*β*42, and tau protein levels, were all based on R software 4.0.3 (R Foundation for Statistical Computing, Vienna, Austria). *P* < 0.05 is considered statistically different.

## 3. Results

### 3.1. Association between Abdominal Obesity and MoCA Scores

#### 3.1.1. Comparison of Basic and Human Body Data

After screening by the eligibility criteria, 196 qualified subjects completed the MoCA-C test. The subjects were divided into abdominal obesity (*n* = 156) and nonabdominal obesity (*n* = 40) groups.

In terms of baseline data, the percentages of age, BMI, NC, WC, HC and the proportion of hypertension in the abdominal obesity group were significantly higher than those in the nonabdominal obesity group (*P* < 0.05) (Table 1). There were no significant differences between gender, the proportion of severe OSAHS, the proportion of diabetes, the proportion of smoking, the proportion of alcohol consumption, the years of education, and the ESS score between the two groups (*P* ≥ 0.05) (Table 1). In terms of PSG indicators, the AHI and ODI indexes of the obesity group were significantly higher than those of the nonabdominal obesity group, while the LSpO2 of the abdominal obesity group was significantly lower (*P* < 0.05) (Table 1).

#### 3.1.2. Comparison of Each Item Score in MoCA

When no factors are corrected, the visuospatial and executive ability, language, and total MoCA scores of the abdominal obesity group were significantly lower than those of the nonabdominal obesity group (3.85 ± 1.02 vs. 4.45 ± 0.68, *P* = 0.001; 1.99 ± 0.68 vs. 2.43 ± 0.68, *P* ≤ 0.001; and 24.53 ± 2.68 vs. 25.65 ± 2.36, *P* = 0.030, respectively). No significant difference was found between the two groups in naming, attention, abstraction, delayed recall, and orientation score (*P* ≥ 0.05) (Table 1). After correcting for gender, age, years of education, ESS, severe OSAHS, smoking and alcohol consumption, there were still significant differences in visuospatial and executive ability, and language scores between the two groups (*P*^1^ = 0.008 and *P*^1^ = 0.004, respectively). And no significant differences in naming, attention, abstraction, delayed recall, orientation total and MoCA scores between the two groups were observed (*P*^1^ ≥ 0.05) (Table 1).

#### 3.1.3. Correlation between WC and the Various Items Scores of MoCA

**Figure 1:**
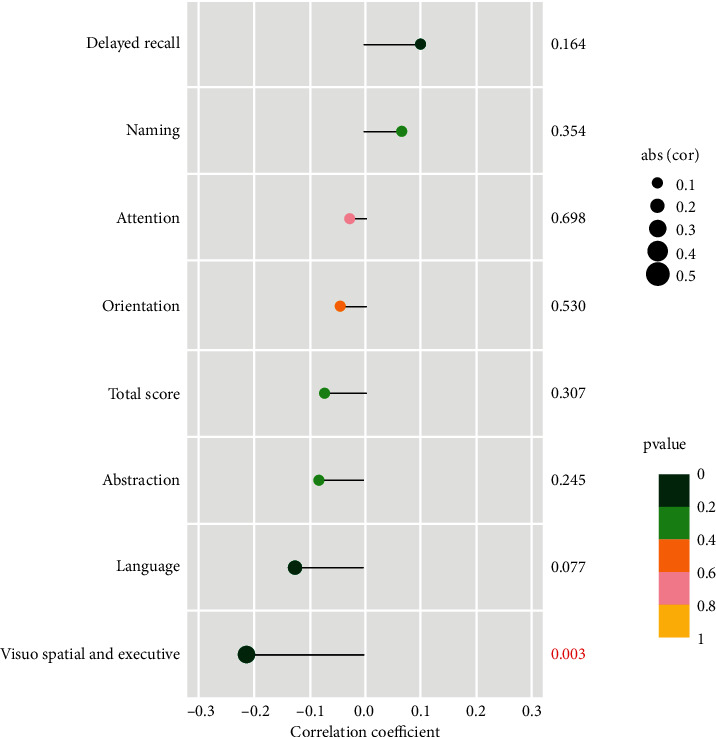
The correlation between WC and the various items of MoCA. The number closely connected to the label of each lollipop shows the corresponding *P* value displayed in different colors. The different sizes of each lollipop ball represent different correlation coefficients.

These results imply that abdominal obesity is associated with cognitive function. Therefore, we further analyzed the correlation between WC and various cognitive functions. The high negative correlation between WC and visuospatial and executive, and language scores further supports the previous conclusions (Figure 1). But unfortunately, only the language score shows significance (*P* < 0.05).

#### 3.1.4. Identification of the Influencing Factors of Some Items Scores in MoCA

Linear regression analysis based on visuospatial and executive, language and total scores, and various potential factors were performed to identify the independent influencing factors of these cognitive functions. Factors used in the linear regression included severe OSAHS, abdominal obesity, gender, age, ESS, smoking, alcohol consumption, and years of education (Table 2).

The independent influencing factors of visuospatial and executive score included abdominal obesity (beta = −0.159, *P* = 0.008) and years of education (beta = −0.523, *P* < 0.001). The only independent influencing factors of language scores was abdominal obesity (beta = −0.201, *P* = 0.004). In addition, severe OSAHS (beta = −0.180, *P* = 0.002), age (beta = −0.261, *P* < 0.001) and years of education (beta = 0.454, *P* < 0.001) were identified as independent influencing factors of the MoCA total score. All the results are shown in Table 2.

### 3.2. Association between Abdominal Obesity and SDMT/TMT Indicators

#### 3.2.1. Comparison of Basic and Human Body Data

In the SDMT and TMT tests, 161 subjects were qualified. The subjects were divided into abdominal obesity (*n* = 126) and nonabdominal obesity (*n* = 35) groups.

In terms of baseline data, the percentages of BMI, NC, WC, HC, and the proportion of hypertension in the abdominal obesity group were significantly higher than those in the nonabdominal obesity group (*P* < 0.05) (Table 3). However, gender, age, the proportion of severe OSAHS, the proportion of diabetes, the proportion of smoking, the proportion of alcohol consumption, years of education, and ESS score were not significantly different between the two groups (*P* ≥ 0.05) (Table 3). In terms of PSG indicators, the AHI and ODI indicators of the abdominal obesity group were significantly higher than those of the nonabdominal obesity group, while the LSpO2 of the abdominal obesity group was significantly lower than that of the nonabdominal obesity group (*P* < 0.05) (Table 3).

#### 3.2.2. Comparison of SDMT/TMT Indicators

When no factors were corrected, there was no significant difference in SDMT and TMT indicators between the two groups (both *P* ≥ 0.05) (Table 3). After correcting for gender, age, years of education, ESS, severe OSAHS, smoking and alcohol consumption, there was still no significant difference in SDMT and TMT indicators between the two groups (*P*^1^ > 0.05) (Table 3).

#### 3.2.3. Correlation between WC and SDMT/TMT Indicators

**Figure 2:**
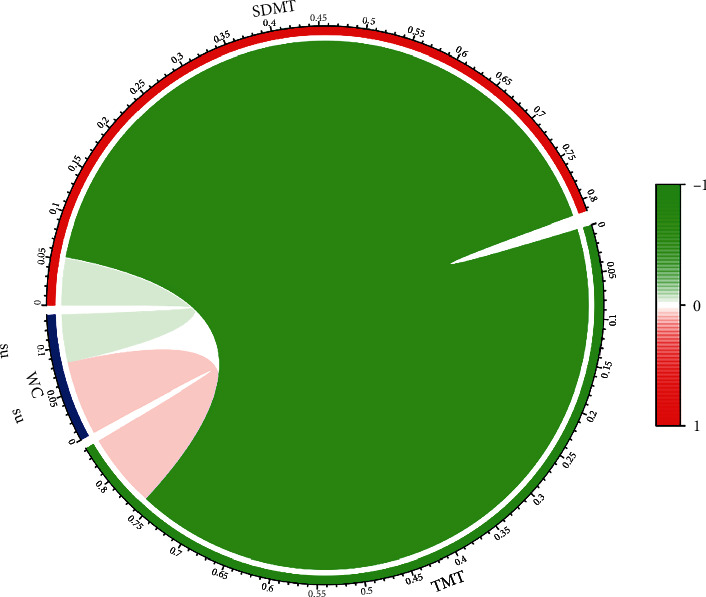
The correlation between WC and SDMT/TMT indicators. The legend of the circle graph shows the correlation coefficients corresponding to different colors. Above the circle: ns: no significance.

Although no significant difference was found, the correlation between WC and SDMT/TMT indicators was still further analyzed. Unfortunately, we still have not observed a significant result (*P* ≥ 0.05, Figure 2).

### 3.3. Association between A*β*40, A*β*42, and Tau Protein Levels and Abdominal Obesity

#### 3.3.1. Comparison of Basic and Human Body Data

The subjects (*n* = 45) were divided into abdominal obesity (*n* = 34) and nonabdominal obesity (*n* = 11). In terms of baseline data, the BMI, NC, WC, and HC of the abdominal obesity group were significantly higher (all *P* < 0.05) (Table 4). However, the proportion of gender, age, the proportion of gender severe OSAHS, the proportion of gender hypertension, the proportion of gender alcohol consumption, the proportion of gender smoking, years of education, and ESS score were not significantly different between the two groups (*P* both ≥0.05) (Table 4). In terms of PSG indicators, AHI, ODI and LSpO2 were not significantly different between the two groups (*P* ≥ 0.05) (Table 4).

#### 3.3.2. Comparison of the Levels of A*β*40, A*β*42, and Tau Protein

**Figure 3:**
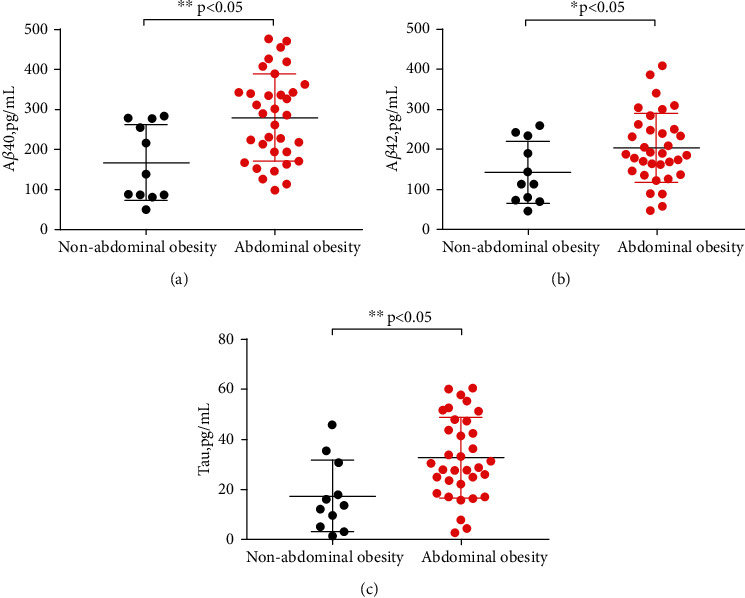
Comparison of A*β*40, A*β*42, and tau protein levels between nonabdominal obesity and abdominal obesity groups, respectively. (a) A*β*40. (b) A*β*42. (c) Tau. Before *P*: ∗ means *P* < 0.05; ∗∗ means *P* < 0.01.

Before and after correcting gender, age, years of education, ESS score, severe OSAHS, smoking and alcohol consumption, the levels of A*β*40, A*β*42, and tau protein in the abdominal obesity group were significantly higher than those in the non-to-moderate OSAHS group (279.47 ± 108.93 vs. 166.98 ± 94.56, *P* = 0.004, *P*^1^ = 0.001; 203.44 ± 86.52 vs. 141.62 ± 77.18, *P* = 0.048, *P*^1^ = 0.006; and 32.62 ± 16.08 vs. 17.29 ± 14.26, *P* = 0.008, *P*^1^ = 0.004, respectively) (Figures 3(a)–3(c)).

#### 3.3.3. Correlation between WC and A*β*40, A*β*42, and Tau Protein Levels

**Figure 4:**
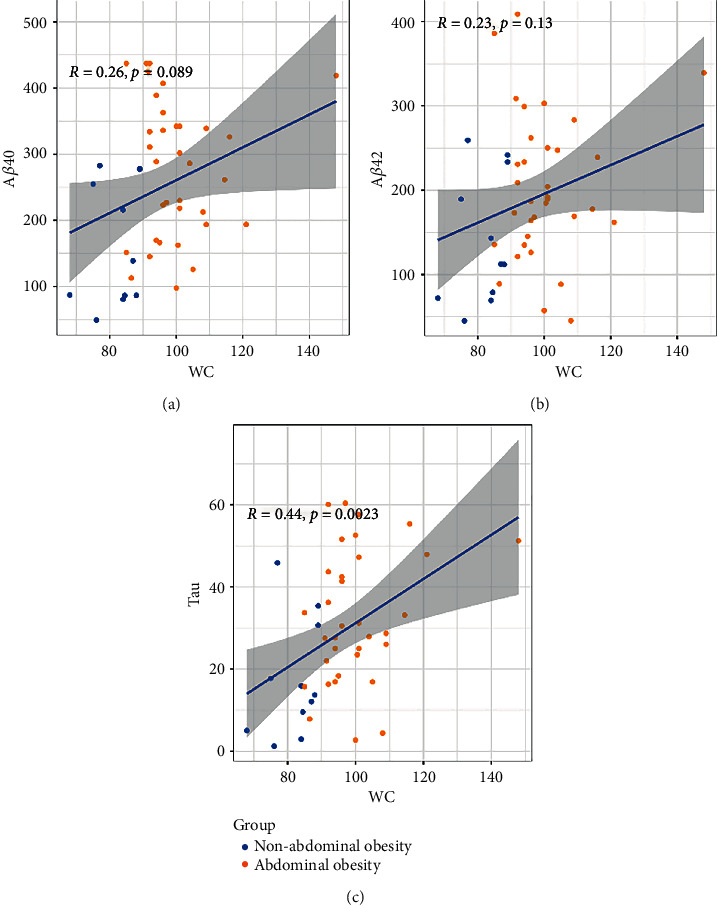
The correlation between WC and A*β*40, A*β*42, and tau protein levels, respectively. (a) A*β*40. (b) A*β*42. (c) Tau. *R* represents the coefficient of correlation.

In Figure 4(c), a significant positive correlation between tau protein level and WC can be observed. Although not significant, a positive correlation trend can still be observed between WC and A*β*40 and A*β*42 protein levels (both *P* ≥ 0.05, Figures 4(a) and 4(b)).

#### 3.3.4. Identification of the Influencing Factors of A*β*40, A*β*42, and Tau Protein Levels

Table 5 shows all results of multiple linear regression based on A*β*40, A*β*42, and tau protein levels and various potential factors. Independent influencing factors of A*β*40 protein level include severe OSAHS (beta = −0.355, *P* = 0.011), abdominal obesity (beta = 0.481, *P* = 0.001) and alcohol consumption (beta = −0.430, *P* = 0.013). Abdominal obesity (beta = 0.410, *P* = 0.006) and smoking (beta = 0.395, *P* = 0.045) were identified as independent influencing factors of A*β*42 protein level. The only independent factor influencing tau protein level was abdominal obesity (beta = 0.450, *P* = 0.004). Surprisingly, abdominal obesity has been identified as an independent factor affecting the levels of all three proteins.

### 3.4. Association between A*β*40, A*β*42 and Tau Protein Levels and Cognitive Functions

**Figure 5:**
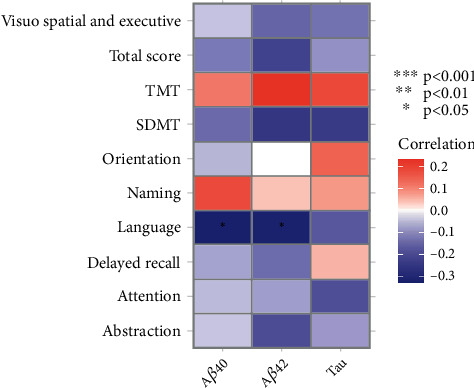
The correlation between A*β*40, A*β*42, and tau protein levels and various cognitive function indicators.

To verify the close correlation between cognitive functions and A*β*40, A*β*42 and tau protein levels, we reran the correlation analysis between them. Unfortunately, only significant negative correlations were observed between A*β*40/A*β*42 protein level and language scores (Figure 5). Even though the other results did not show significance, they still provided a lot of valuable information. Except for naming and TMT, the level of A*β*40 protein is negatively correlated with other project indicators. Except for naming, orientation and TMT, the level of A*β*42 protein is also negatively associated with other project indicators. In addition, TMT indicator has been observed positively correlated with the three protein levels. The opposite results were found in the SDMT indicator.

## 4. Discussion

Cognition is the intelligent process of body recognition and knowledge acquisition. It involves a series of psychological and social behaviors, such as learning, memory, language, thinking, spirit, and emotion. Executive function refers to a person's ability to respond adaptively to situations and successfully engage in independent, purposeful, and self-service behaviors, the foundation of many cognitive, social, and emotional skills [41].

Because obesity is closely related to OSAHS [42], about 40% to 70% of obese people are diagnosed with OSAHS [43, 44]. Obesity is considered one of the most critical risk factors for OSAHS [45]. In addition, through a meta-analysis of previous research systems, Bucks et al. found that most studies support OSAHS patients' deficits in attention/alertness, delayed long-term visual and language memory, visuospatial/structural abilities, and deficits in executive function [46]. Therefore, it is necessary to correct OSAHS, an important factor of cognitive function. To reduce the impact of other potential influencing factors on the research results, we also performed corrections for cognitive impairment risk factors (including age, gender, abdominal obesity, smoking, alcohol consumption, years of education, and ESS score) [47].

Before and after correcting the related influencing factors, lower visual space and execution and language scores were found in the abdominal obesity group, which was also supported by the negative correlation between WC and these two MoCA scores in our study. Conversely, A*β*40, A*β*42, and tau protein levels in the abdominal obesity group were higher than those in the nonabdominal obesity group, also supported by the corresponding correlation results. Not only that, abdominal obesity has also been identified as an independent negative factor of visual space and execution and language scores, as well as an independent positive factor of A*β*40, A*β*42, and tau protein levels in further regression analysis. The above results all imply that abdominal obesity may significantly negatively affect the visual space and execution and language ability of the subject and increase the A*β*40, A*β*42, and tau protein levels of the subject. Our regression analysis also found that age negatively affects delayed recall, overall cognitive attention, and information processing speed. The years of education have also been found to be a positive factor in visual space and execution, abstraction, overall cognition, attention, and information processing speed capabilities.

Few studies focus on the relationship between obesity and cognitive function. It is generally believed that obese subjects usually exhibit deficits in memory, learning, and executive function in the previous study. Moreover, various indicators of obesity, including BMI, WHR, and WC, have also been shown to impair overall cognitive function, learning, memory, and language ability [48]. This study explored the influence of abdominal obesity judged by WC on the cognitive field, which has carried out a deeper exploration in related fields. The worse visual space and executive and language skills observed in the abdominal obesity group supported the views in the literature. In addition to obesity and OSAHS, normal human aging can also change some areas of cognition, such as processing speed, attention, context, and working memory, and rarely affects language ability and recognition memory [49]. Our results are also consistent with previous research results. With age, performance in the areas of delayed recall, overall cognition, attention, and information processing speed decreases.

At present, inflammation, gut-brain axis, and insulin resistance are considered the main mechanisms of obesity in impairing cognitive function. Inflammation is the ultimate common pathway of these mechanisms [26, 50]. First, a long-term high-fat diet can cause increased proinflammatory and inflammatory cytokines in the blood [51]. These factors can increase blood-brain barrier penetration and transport dysfunction [52]. The lesions of the blood-brain barrier directly affect the lateral hypothalamic nucleus and the preoptic area to produce behavioral and mental abnormalities [53] and affect the dorsal hippocampus to cause learning and memory decline [54]. After inflammatory factors enter the brain through the blood-brain barrier, they can further induce a series of inflammatory damage and apoptosis of different types of nerve cells to affect cognitive function [55]. The brain-gut axis mainly affects cognitive function through immune activation, intestinal permeability, intestinal endocrine, and neural signal pathways. Dietary saturated fatty acids can activate Toll-like receptors (TLR) expressed in the intestinal epithelium and innate immune cells [56]. TLR2 activates the conduction cascade to amplify the inflammatory response [57, 58], and TLR4 activates the upregulation of proinflammatory cytokines to trigger brain inflammation [59]. Intestinal flora imbalance may also affect brain development and plasticity by affecting neurotrophins (BDNF) and neurotransmitters (5HT, GABA, etc.), thereby impairing cognitive functions such as memory. Finally, insulin plays an active role in cognition and memory as a neuromodulator and neuroprotective agent in the brain [60, 61]. Excessive free fatty acids in obesity can increase the release of proinflammatory cytokines in the blood [62]. These cytokines not only activate the phosphorylation of insulin receptor substrate (insulin receptor substrate-1 (IRS-1)) at the serine site (normally the tyrosine site) but also activates other inflammation-related negative regulators (such as inhibitors of cytokine signal transduction) in the IRS protein to inhibit cell insulin signal transduction in target organs and cause insulin resistance [63]. In addition, obesity-induced oxidative stress and hyperglycemia lead to mitochondrial dysfunction, generating peripheral insulin resistance [64, 65]. Peripheral insulin resistance can also induce brain inflammation, oxidative stress, and insulin resistance through ceramide production in the liver, leading to neurodegeneration and cognitive impairment [66]. These potential biological processes may explain the worse visual space, execution, and language ability in subjects with abdominal obesity.

Tau protein, a kind of microtubule-associated protein, is necessary for normal neuronal activity [19]. However, under pathological conditions, certain phosphorous sites of tau protein can bind to phosphoric acid and undergo abnormal hyperphosphorylation to form p-tau protein. The p-tau protein makes the microtubule-binding region self-associate and causes abnormal entanglement of microtubule spiral filaments, forming neurofibrillary tangles [18]. The function of A*β* is to maintain neuron growth, synaptic activity, and survival at low levels [20]. However, at sufficiently high levels, A*β* forms aggregates. Eventually, nerve fiber plaques are formed [22]. The systemic inflammation of obesity can promote the production of A*β*, which may be one of the pathogenesis of AD. Peripheral insulin resistance and hyperinsulinemia will increase peripheral free fatty acid levels, and peripheral and central TNF-*α* levels will also increase, thereby reducing and clearing A*β* through the liver, leading to increased peripheral A*β* levels [67, 68]. High levels of A*β* in plasma interfere with the transfer of A*β* from the brain to the periphery, thereby increasing its transfer to the brain. As a result, the release of A*β* from nerve cells is inhibited. The decrease in insulin-degrading enzyme levels in obese individuals also aggravated the deposition of A*β* in neurons. Therefore, accumulation results from a variety of pathological mechanisms, such as increased A*β* production, peripheral clearance dysfunction, and increased brain inflammation, may lead to memory deficits and even AD. Perhaps these underlying processes can explain the upregulation of A*β*40, A*β*42, and tau protein levels in subjects with abdominal obesity and the negative correlation between various cognitive functions and these protein levels observed in our research.

Although not many significant results have been found, there is no doubt that we have expanded the few researches in the relevant fields. Considering that our research is based on large sample testing, highly sensitive and specific protein detection technology (RIA), and multiple and complex analysis methods, the results obtained have a high degree of credibility. But unfortunately, the limited number of blood samples could easily amplify the chance of type II error and lead to inaccurate results. The shortcomings of the cross-sectional study and the limited data sources may cause some deficiencies in this study. As a commonly used scale, MoCA still has many shortcomings, such as limited cognitive domains and poor specificity and sensitivity, leading to deviations in our results. Although many subjects were recruited in our study, more significant and reliable results still require a larger sample size to support. Although some factors that may affect cognitive function have been adjusted, it is difficult for us to correct the interference of other confounding factors, such as APOE4 genotype and tester's experience. Due to the limitations of clinical conditions, this study failed to collect more sensitive cerebrospinal fluid to determine related protein levels. Limited experimental conditions also limit us to explore deeper mechanisms combined with more basic experiments.

## Figures and Tables

**Table 1:** Comparison of baseline characteristics, PSG indicators, and MoCA scores between the nonabdominal obesity and the abdominal obesity groups.

	Nonabdominal obesity (*n* = 40)	Abdominal obesity (*n* = 156)	*P*	*P* ^1^
Gender (female/male)	36/4	137/19	0.085	—
Age (years)	39.75 ± 14.00	43.58 ± 11.36	0.048	—
BMI (kg/m^2^)	23.40 ± 2.14	27.86 ± 3.63	0	—
Neck circumference (cm)	37.34 ± 2.50	41.00 ± 3.39	0	—
Waist circumference (cm)	83.04 ± 6.81	99.99 ± 9.15	0	—
Hip circumference (cm)	95.38 ± 4.51	102.71 ± 10.08	0	—
Severe OSAHS, no. (%)	19 (47.5%)	100 (64.1%)	0.055	—
Hypertension, no. (%)	5 (12.5%)	50 (32.1%)	0.014	—
Diabetes, no. (%)	0 (0.0%)	7 (4.5%)	0.348	—
Smoking, no. (%)	18 (45.0%)	75 (48.1%)	0.728	—
Alcohol consumption, no. (%)	23 (57.5%)	73 (46.8%)	0.227	—
Education (years)	12.15 ± 3.77	10.98 ± 3.65	0.055	—
ESS score	9.15 ± 4.59	10.60 ± 5.32	0.102	—
PSG indicator				
AHI	29.47 ± 22.11	44.46 ± 27.60	0.002	—
ODI	25.35 ± 22.80	44.65 ± 30.41	0	—
LSpO2	80.98 ± 9.86	73.54 ± 13.61	0.003	—
MoCA score				
Visuospatial and executive	4.45 ± 0.68	3.85 ± 1.02	0.001	0.008
Naming	2.90 ± 0.30	2.92 ± 0.31	0.45	0.322
Attention	5.87 ± 0.56	5.88 ± 0.51	0.674	0.698
Language	2.43 ± 0.68	1.99 ± 0.68	0	0.004
Abstraction	1.63 ± 0.54	1.49 ± 0.74	0.553	0.947
Delayed recall	2.48 ± 1.41	2.55 ± 1.45	0.85	0.136
Orientation	5.90 ± 0.30	5.85 ± 0.41	0.605	0.667
Total score	25.65 ± 2.36	24.53 ± 2.68	0.03	0.374

*P* value: comparison of baseline characteristics, PSG indicators, and MoCA scores, without correcting any factors; *P*^1^ value: comparison of the various items and total scores of MoCA score, correcting for the factors of gender, age, years of education, ESS, severe OSAHS, smoking, and alcohol consumption. BMI: body mass index; OSAHS: obstructive sleep apnea-hypopnea syndrome; ESS: Epworth Sleepiness Scale; PSG: polysomnography; AHI: apnea-hypopnea index; ODI: oxygen-desaturation index; LSpO2: lowest oxygen saturation; MoCA: Montreal Cognitive Assessment.

**Table 2:** Linear regression analysis based on various potential influencing factors and MoCA scores of some items.

		Severe OSAHS	Abdominal obesity	Gender	Age	ESS	Smoking	Alcohol consumption	Education
Visuospatial and executive	Beta	-0.070	-0.159	-0.032	-0.063	-0.029	0.014	-0.003	0.523
	*P*	0.265	0.008	0.618	0.305	0.643	0.837	0.962	<0.001
Language	Beta	-0.048	-0.201	-0.092	-0.141	-0.092	-0.004	-0.050	0.140
	*P*	0.506	0.004	0.223	0.051	0.213	0.961	0.526	0.071
Total score	Beta	-0.180	-0.050	-0.073	-0.261	-0.044	-0.047	-0.040	0.454
	*P*	0.002	0.374	0.225	<0.001	0.462	0.471	0.537	<0.001

ESS: Epworth Sleepiness Scale.

**Table 3:** Comparison of baseline characteristics, PSG indicators, SDMT, and TMT between nonabdominal obesity and abdominal obesity groups.

	Nonabdominal obesity (*n* = 35)	Abdominal obesity (*n* = 126)	*P*	*P* ^1^
Gender (female/male)	32/3	109/17	0.569	—
Age (years)	40.71 ± 14.80	43.44 ± 11.56	0.171	—
BMI (kg/m^2^)	22.48 ± 2.08	27.93 ± 3.66	0	—
Neck circumference (cm)	36.69 ± 2.43	41.07 ± 3.40	0	—
Waist circumference (cm)	81.71 ± 7.15	99.75 ± 9.01	0	—
Hip circumference (cm)	93.83 ± 5.27	102.43 ± 10.63	0	—
Severe OSAHS, no. (%)	17 (48.6%)	81 (64.3%)	0.092	—
Hypertension, no. (%)	1 (2.9%)	30 (23.8%)	0.005	—
Diabetes, no. (%)	0 (0.0%)	2 (1.6%)	1	—
Smoking, no. (%)	17 (48.6%)	69 (54.8%)	0.516	—
Alcohol consumption, no. (%)	19 (54.3%)	51 (40.5%)	0.145	—
Education (years)	11.13 ± 3.67	10.13 ± 3.33	0.126	—
ESS score	9.26 ± 4.38	10.71 ± 4.87	0.091	—
PSG indicator				
AHI	28.89 ± 21.92	45.03 ± 28.43	0.003	—
ODI	23.30 ± 23.13	45.38 ± 31.70	0	—
LSpO2	81.97 ± 10.37	73.51 ± 13.22	0.001	—
Attention and information processing speed				
SDMT (correct number)	48.46 ± 12.62	44.99 ± 15.28	0.256	0.808
TMT (time consumption)	42.26 ± 13.36	49.11 ± 21.66	0.144	0.467

*P* value: compare baseline characteristics and PSG, SDMT, and TMT indicators, without correcting any factors; *P*^1^ value: comparing SDMT and TMT indicators, correcting for the gender, age, years of education, ESS, severe OSAHS, smoking, and alcohol consumption factors. BMI: body mass index; OSAHS: obstructive sleep apnea-hypopnea syndrome; ESS: Epworth Sleepiness Scale; PSG: polysomnography; AHI: apnea-hypopnea index; ODI: oxygen-desaturation index; LSpO2: lowest oxygen saturation; MoCA: Montreal Cognitive Assessment; SDMT: Symbol Digit Modalities Test; TMT: Trail Making Test.

**Table 4:** Comparison of baseline characteristics, PSG indicator, and protein levels between nonabdominal obesity and abdominal obesity groups.

	Nonabdominal obesity (*n* = 11)	Abdominal obesity (*n* = 34)	*P*	*P* ^1^
Gender (female/male)	8/3	26/8	1	—
Age (years)	48.18 ± 18.79	40.27 ± 13.51	0.161	—
BMI (kg/m^2^)	22.44 ± 2.61	29.31 ± 3.91	0	—
Neck circumference (cm)	36.14 ± 2.65	40.82 ± 3.49	0	—
Waist circumference (cm)	81.96 ± 6.92	100.12 ± 11.97	0	—
Hip circumference (cm)	90.64 ± 7.72	102.00 ± 18.12	0	—
Severe OSAHS, no. (%)	3 (27.3%)	16 (47.1%)	0.309	—
Hypertension, no. (%)	0 (0.0%)	7 (20.6%)	0.168	—
Diabetes, no. (%)	0 (0.0%)	0 (0.0%)	—	—
Smoking, no. (%)	7 (63.6%)	14 (41.2%)	0.194	—
Alcohol consumption, no. (%)	7 (63.6%)	11 (32.4%)	0.086	—
Education (years)	7.64 ± 4.06	9.82 ± 3.86	0.118	—
ESS score	10.18 ± 4.02	10.71 ± 4.09	0.73	—
PSG indicator				
AHI	20.53 ± 23.71	35.86 ± 33.06	0.154	—
ODI	19.55 ± 24.45	35.54 ± 35.76	0.166	—
LSpO2	81.16 ± 14.62	75.24 ± 14.99	0.149	—
Protein level				
A*β*40	166.98 ± 94.56	279.47 ± 108.93	0.004	0.001
A*β*42	141.62 ± 77.18	203.44 ± 86.52	0.048	0.006
Tau	17.29 ± 14.26	32.62 ± 16.08	0.008	0.004

*P* value: compare baseline characteristics, PSG indicator, and A*β*40, A*β*42, and tau protein levels, without correcting any factors; *P*^1^ value: compare protein levels, correcting for gender, age, years of education, ESS, severe OSAHS, smoking, and alcohol consumption factors. BMI: body mass index; OSAHS: obstructive sleep apnea-hypopnea syndrome; ESS: Epworth Sleepiness Scale; PSG: polysomnography; AHI: apnea-hypopnea index; ODI: oxygen-desaturation index; LSpO2: lowest oxygen saturation.

**Table 5:** Linear regression analysis based on A*β*40, A*β*42, and tau protein levels and various potential influencing factors.

		Severe OSAHS	Abdominal obesity	Gender	Age	ESS	Smoking	Alcohol consumption	Education
A*β*40	Beta	-0.355	0.481	-0.316	0.149	-0.149	0.343	-0.43	-0.074
	*P*	0.011	0.001	0.06	0.294	0.279	0.058	0.013	0.621
A*β*42	Beta	-0.198	0.41	-0.236	0.222	-0.047	0.395	-0.27	-0.099
	*P*	0.176	0.006	0.191	0.154	0.753	0.045	0.141	0.546
Tau	Beta	-0.09	0.45	-0.288	0.308	0.058	0.042	-0.222	-0.154
	*P*	0.547	0.004	0.123	0.058	0.703	0.831	0.238	0.36

ESS: Epworth Sleepiness Scale.

## Data Availability

The data in this study were from the Department of Otolaryngology Head and Neck Surgery, The First Affiliated Hospital of Nanchang University.

## Abbreviations

CHNS: The China Health and Nutrition Survey
WC: Waist circumference
WHR: Waist-to-hip ratio
AD: Alzheimer disease
A*β*: Amyloid *β*-protein
MCI: Mild cognitive impairment
NC: Neck circumference
HC: Hip circumference
OSAHS: Obstructive sleep apnea-hypopnea syndrome
PSG: Polysomnography
AHI: Apnea-hypopnea index
ODI: Oxygen-desaturation index
LSpO2: Lowest oxygen saturation
ESS: Epworth Sleepiness Scale
MoCA-C: Chinese-Beijing Version of Montreal Cognitive Assessment
SDMT: Symbol Digit Modalities Test
TMT-A: Trail Making Test-Part A
RIA: Radioimmunoassay
TLR: Toll-like receptors
DAMPs: Damage-related molecular patterns
BDNF: Neurotrophins.

## References

[1] The GBD 2015 Obesity Collaborators (2017). Health effects of overweight and obesity in 195 countries over 25 years. *The New England Journal of Medicine*.

[2] World Health Organization Obesity and overweight. https://www.who.int/en/newsroom/fact-sheets/detail/obesity-and-overweight.

[3] Gondim P. N., Rosa P. V., Okamura D. (2018). Benefits of fish oil consumption over other sources of lipids on metabolic parameters in obese rats. *Nutrients*.

[4] Hu L., Huang X., You C. (2017). Prevalence of overweight, obesity, abdominal obesity and obesity-related risk factors in southern China. *PLoS One*.

[5] Harper R. M., Kumar R., Macey P. M., Woo M. A., Ogren J. A. (2014). Affective brain areas and sleep-disordered breathing. *Progress in Brain Research*.

[6] Kopelman P. G. (2000). Obesity as a medical problem. *Nature*.

[7] Lawrence V. J., Kopelman P. G. (2004). Medical consequences of obesity. *Clinics in Dermatology*.

[8] Bray G. A. (2004). Medical consequences of obesity. *The Journal of Clinical Endocrinology and Metabolism*.

[9] Oh T. J., Moon J. H., Choi S. H. (2019). Body-weight fluctuation and incident diabetes mellitus, cardiovascular disease, and mortality: a 16-year prospective cohort study. *The Journal of Clinical Endocrinology and Metabolism*.

[10] Reis J. P., Macera C. A., Araneta M. R., Lindsay S. P., Marshall S. J., Wingard D. L. (2009). Comparison of overall obesity and body fat distribution in predicting risk of mortality. *Obesity*.

[11] Anari R., Amani R., Latifi S. M., Veissi M., Shahbazian H. (2017). Association of obesity with hypertension and dyslipidemia in type 2 diabetes mellitus subjects. *Diabetes and Metabolic Syndrome: Clinical Research and Reviews*.

[12] Rolls A., Colas D., Adamantidis A. (2011). Optogenetic disruption of sleep continuity impairs memory consolidation. *Proceedings of the National Academy of Sciences of the United States of America*.

[13] Naismith S., Winter V., Gotsopoulos H., Hickie I., Cistulli P. (2004). Neurobehavioral functioning in obstructive sleep apnea: differential effects of sleep quality, hypoxemia and subjective sleepiness. *Journal of Clinical and Experimental Neuropsychology*.

[14] Tartar J. L., Ward C. P., McKenna J. T. (2006). Hippocampal synaptic plasticity and spatial learning are impaired in a rat model of sleep fragmentation. *The European Journal of Neuroscience*.

[15] Barnes D. E., Yaffe K. (2011). The projected effect of risk factor reduction on Alzheimer's disease prevalence. *The Lancet*.

[16] Aslan A. K. D., Starr J. M., Pattie A., Deary I. (2015). Cognitive consequences of overweight and obesity in the ninth decade of life?. *Age and Ageing*.

[17] Albanese E., Davis B., Jonsson P. V. (2015). Overweight and obesity in midlife and brain structure and dementia 26 years later: the AGES-Reykjavik study. *American Journal of Epidemiology*.

[18] Aribisala B. S., Valdés Hernández M. C., Royle N. A. (2013). Brain atrophy associations with white matter lesions in the ageing brain: the Lothian Birth Cohort 1936. *European Radiology*.

[19] Harada A., Oguchi K., Okabe S. (1994). Altered microtubule organization in small-calibre axons of mice lacking *tau* protein. *Nature*.

[20] Parihar M. S., Brewer G. J. (2010). Amyloid-*β* as a modulator of synaptic plasticity. *Journal of Alzheimer's Disease*.

[21] Baril A.-A., Carrier J., Lafrenière A. (2018). Biomarkers of dementia in obstructive sleep apnea. *Sleep Medicine Reviews*.

[22] Daulatzai M. A. (2015). Evidence of neurodegeneration in obstructive sleep apnea: relationship between obstructive sleep apnea and cognitive dysfunction in the elderly. *Journal of Neuroscience Research*.

[23] Xie L., Kang H., Xu Q. (2013). Sleep drives metabolite clearance from the adult brain. *Science*.

[24] Ju Y.-E. S., Ooms S. J., Sutphen C. (2017). Slow wave sleep disruption increases cerebrospinal fluid amyloid-*β* levels. *Brain*.

[25] (2021). 2021 Alzheimer's disease facts and figures. *Alzheimer's & Dementia*.

[26] Solas M., Milagro F. I., Ramírez M. J., Martínez J. A. (2017). Inflammation and gut-brain axis link obesity to cognitive dysfunction: plausible pharmacological interventions. *Current Opinion in Pharmacology*.

[27] Jung Y. H., Park S., Jang H. (2020). Frontal-executive dysfunction affects dementia conversion in patients with amnestic mild cognitive impairment. *Scientific Reports*.

[28] Coisne C., Engelhardt B. (2011). Tight junctions in brain barriers during central nervous system inflammation. *Antioxidants & Redox Signaling*.

[29] Smith E., Hay P., Campbell L., Trollor J. N. (2011). A review of the association between obesity and cognitive function across the lifespan: implications for novel approaches to prevention and treatment. *Obesity Reviews*.

[30] Loef M., Walach H. (2013). Midlife obesity and dementia: meta-analysis and adjusted forecast of dementia prevalence in the United States and China. *Obesity*.

[31] Xu W. L., Atti A. R., Gatz M., Pedersen N. L., Johansson B., Fratiglioni L. (2011). Midlife overweight and obesity increase late-life dementia risk: a population-based twin study. *Neurology*.

[32] Chen X., Zhang R., Xiao Y., Dong J., Niu X., Kong W. (2015). Reliability and validity of the Beijing version of the Montreal Cognitive Assessment in the evaluation of cognitive function of adult patients with OSAHS. *PLoS One*.

[33] Aaron S. (1982). *Symbol Digit Modalities Test (SDMT)*.

[34] Levin H. S., Li X., McCauley S. R., Hanten G., Wilde E. A., Swank P. (2013). Neuropsychological outcome of mTBI: a principal component analysis approach. *Journal of Neurotrauma*.

[35] Bowie C. R., Harvey P. D. (2006). Administration and interpretation of the Trail Making Test. *Nature Protocols*.

[36] Hodge M. A. R., Siciliano D., Withey P. (2010). A randomized controlled trial of cognitive remediation in schizophrenia. *Schizophrenia Bulletin*.

[37] (2007). Chinese guidelines on prevention and treatment of dyslipidemia in adults. *Zhonghua Xin Xue Guan Bing Za Zhi*.

[38] Malhotra R. K., Kirsch D. B., Kristo D. A. (2018). Polysomnography for obstructive sleep apnea should include arousal-based scoring: an American Academy of Sleep Medicine position statement. *Journal of Clinical Sleep Medicine*.

[39] Berry R. B., Budhiraja R., Gottlieb D. J., Gozal D., Iber C., Kapur V. K. (2012). Rules for scoring respiratory events in sleep: update of the 2007 AASM Manual for the Scoring of Sleep and Associated Events. Deliberations of the Sleep Apnea Definitions Task orce of the American Academy of Sleep Medicine. *Journal of Clinical Sleep Medicine*.

[40] Alterki A., Joseph S., Thanaraj T. A. (2020). Targeted metabolomics analysis on obstructive sleep apnea patients after multilevel sleep surgery. *Metabolites*.

[41] Saunamäki T., Himanen S.-L., Polo O., Jehkonen M. (2009). Executive dysfunction in patients with obstructive sleep apnea syndrome. *European Neurology*.

[42] Gami A. S., Caples S. M., Somers V. K. (2003). Obesity and obstructive sleep apnea. *Endocrinology and Metabolism Clinics of North America*.

[43] Young T., Finn L., Peppard P. E. (2008). Sleep disordered breathing and mortality: eighteen-year follow-up of the Wisconsin sleep cohort. *Sleep*.

[44] Lopez P. P., Stefan B., Schulman C. I., Byers P. M. (2008). Prevalence of sleep apnea in morbidly obese patients who presented for weight loss surgery evaluation: more evidence for routine screening for obstructive sleep apnea before weight loss surgery. *The American Surgeon*.

[45] Ievers-Landis C. E., Redline S. (2007). Pediatric sleep apnea: implications of the epidemic of childhood overweight. *American Journal of Respiratory and Critical Care Medicine*.

[46] Bucks R. S., Olaithe M., Eastwood P. (2013). Neurocognitive function in obstructive sleep apnoea: a meta-review. *Respirology*.

[47] Lal C., Strange C., Bachman D. (2012). Neurocognitive impairment in obstructive sleep apnea. *Chest*.

[48] Gunstad J., Lhotsky A., Wendell C. R., Ferrucci L., Zonderman A. B. (2010). Longitudinal examination of obesity and cognitive function: results from the Baltimore longitudinal study of aging. *Neuroepidemiology*.

[49] Mathieu A., Mazza S., Décary A. (2008). Effects of obstructive sleep apnea on cognitive function: a comparison between younger and older OSAS patients. *Sleep Medicine*.

[50] Leigh S.-J., Morris M. J. (2020). Diet, inflammation and the gut microbiome: mechanisms for obesity-associated cognitive impairment. *Biochimica et Biophysica Acta - Molecular Basis of Disease*.

[51] Alosco M. L., Gunstad J. (2014). The negative effects of obesity and poor glycemic control on cognitive function: a proposed model for possible mechanisms. *Current Diabetes Reports*.

[52] Obermeier B., Daneman R., Ransohoff R. M. (2013). Development, maintenance and disruption of the blood-brain barrier. *Nature Medicine*.

[53] Thaler J. P., Yi C.-X., Schur E. A. (2012). Obesity is associated with hypothalamic injury in rodents and humans. *The Journal of Clinical Investigation*.

[54] Kanoski S. E., Grill H. J. (2017). Hippocampus contributions to food intake control: mnemonic, neuroanatomical, and endocrine mechanisms. *Biological Psychiatry*.

[55] Moraes J. C., Coope A., Morari J. (2009). High-fat diet induces apoptosis of hypothalamic neurons. *PLoS One*.

[56] Hwang D. H., Kim J.-A., Lee J. Y. (2016). Mechanisms for the activation of Toll-like receptor 2/4 by saturated fatty acids and inhibition by docosahexaenoic acid. *European Journal of Pharmacology*.

[57] Hayward J. H., Lee S. J. (2014). A decade of research on TLR2 discovering its pivotal role in glial activation and neuroinflammation in neurodegenerative diseases. *Experimental Neurobiology*.

[58] Kumar V. (2019). Toll-like receptors in the pathogenesis of neuroinflammation. *Journal of Neuroimmunology*.

[59] Guillemot-Legris O., Muccioli G. G. (2017). Obesity-induced neuroinflammation: beyond the hypothalamus. *Trends in Neurosciences*.

[60] Blázquez E., Velázquez E., Hurtado-Carneiro V., Ruiz-Albusac J. M. (2014). Insulin in the brain: its pathophysiological implications for states related with central insulin resistance, type 2 diabetes and Alzheimer’s disease. *Frontiers in Endocrinology*.

[61] Soto M., Cai W., Konishi M., Kahn C. R. (2019). Insulin signaling in the hippocampus and amygdala regulates metabolism and neurobehavior. *Proceedings of the National Academy of Sciences of the United States of America*.

[62] Osborn O., Olefsky J. M. (2012). The cellular and signaling networks linking the immune system and metabolism in disease. *Nature Medicine*.

[63] Werner E. D., Lee J., Hansen L., Yuan M., Shoelson S. E. (2004). Insulin resistance due to phosphorylation of insulin receptor substrate-1 at serine 302. *The Journal of Biological Chemistry*.

[64] Boden G. (2011). Obesity, insulin resistance and free fatty acids. *Current Opinion in Endocrinology, Diabetes, and Obesity*.

[65] Peng Y., Liu J., Shi L. (2016). Mitochondrial dysfunction precedes depression of AMPK/AKT signaling in insulin resistance induced by high glucose in primary cortical neurons. *Journal of Neurochemistry*.

[66] Górski J. (2012). Ceramide and insulin resistance: how should the issue be approached?. *Diabetes*.

[67] Busquets O., Ettcheto M., Pallàs M. (2017). Long-term exposition to a high fat diet favors the appearance of *β*-amyloid depositions in the brain of C57BL/6J mice. A potential model of sporadic Alzheimer's disease. *Mechanisms of Ageing and Development*.

[68] Lourenco M. V., Clarke J. R., Frozza R. L. (2013). TNF-*α* mediates PKR-dependent memory impairment and brain IRS-1 inhibition induced by Alzheimer's *β*-amyloid oligomers in mice and monkeys. *Cell Metabolism*.

